# Primary care transformation in Scotland: a qualitative evaluation of the views of patients

**DOI:** 10.3399/BJGP.2023.0437

**Published:** 2024-05-14

**Authors:** Eddie Donaghy, Kieran Sweeney, David Henderson, Colin Angus, Morag Cullen, Mary Hemphill, Harry HX Wang, Bruce Guthrie, Stewart W Mercer

**Affiliations:** Centre for Population Health Studies, Usher Institute, College of Medicine and Veterinary Medicine, University of Edinburgh, Edinburgh, UK;; Centre for Population Health Studies, Usher Institute, College of Medicine and Veterinary Medicine, University of Edinburgh, Edinburgh, UK;; Centre for Population Health Studies, Usher Institute, College of Medicine and Veterinary Medicine, University of Edinburgh, Edinburgh, UK;; Centre for Population Health Studies, Usher Institute, College of Medicine and Veterinary Medicine, University of Edinburgh, Edinburgh, UK;; Centre for Population Health Studies, Usher Institute, College of Medicine and Veterinary Medicine, University of Edinburgh, Edinburgh, UK;; Centre for Population Health Studies, Usher Institute, College of Medicine and Veterinary Medicine, University of Edinburgh, Edinburgh, UK;; School of Public Health, Sun Yat-Sen University, Guangzhou, China.; Centre for Population Health Studies, Usher Institute, College of Medicine and Veterinary Medicine, University of Edinburgh, Edinburgh, UK;; Centre for Population Health Studies, Usher Institute, College of Medicine and Veterinary Medicine, University of Edinburgh, Edinburgh, UK;

**Keywords:** primary care, health care reform, GP contracts, qualitative research, multimorbidity, social deprivation

## Abstract

**Background:**

The new Scottish GP contract introduced in April 2018 aims to improve quality of care through expansion of the multidisciplinary team (MDT) to enable GPs to spend more time as expert medical generalists with patients with complex needs.

**Aim:**

To explore patients’ views on the changes in general practice in Scotland since the inception of the new contract.

**Design and setting:**

Qualitative study with 30 patients (10 living in urban deprived areas, 10 living in urban affluent/mixed urban areas, and 10 living in remote and rural areas).

**Method:**

In-depth semi-structured interviews with thematic analysis.

**Results:**

Patients were generally unaware of the new GP contract, attributing recent changes in general practice to the COVID-19 pandemic. Ongoing concerns included access to GP consultations (especially face-to-face ones), short consultation length with GPs, and damage to continuity of care and the GP–patient relationship. Most patients spoke positively about consultations with MDT staff but still wanted to see a known GP for health concerns that they considered potentially serious. These issues were especially concerning for patients with multiple complex problems, particularly those from deprived areas.

**Conclusion:**

Following the introduction of the new Scottish GP contract, patients in this study’s sample were accepting of first contact care from the MDT but still wanted continuity of care and longer face-to-face consultations with GPs. These findings suggest that the expert generalist role of the GP is not being adequately supported by the new contract, especially in deprived areas, though further quantitative research is required to confirm this.

## Introduction

The Scottish Government has a vision to transform primary care to help address the challenges of the ageing population and health inequalities.^1^^–^^3^ They introduced a new Scottish GP contract in April 2018^4^ following the abolition of the Quality and Outcomes Framework in April 2016.^5^ The stated aims of the 2018 contract were:

- to improve access for patients, address health inequalities, and improve population health, including mental health,
- to provide financial stability for GPs, and reduce GP workload through the expansion of the primary care multidisciplinary team (MDT),
- to redefine the role of the GP as an expert medical generalist focusing on complex care.

Between March 2018 and March 2022, 3220 whole-time equivalent new MDT staff were appointed in primary care, with the largest group being pharmacists/pharmacy technicians^6^ with a further increase of 518 whole-time equivalent between March 2022 and March 2023.^7^ The authors’ previous qualitative evaluation with GPs and MDT staff, conducted in 2022 and published in this journal, indicated many ongoing challenges to effective implementation.^8^

These efforts to transform primary care in Scotland to better meet the needs of the population are in line with approaches taken by many other countries.^9^ Although multiple approaches are being taken to primary care transformation, expansion of the MDT is the most common in high-income countries and China,^9^ and is specifically encouraged by the World Health Organization.^10^ It has been noted that patients’ views are rarely included in large scale reforms of primary care,^9^^,^^11^ with little attention paid to health inequalities and the needs of older patients.^9^

Despite the radical nature of the reforms in general practice in Scotland, there appears to have been very limited evaluation of patients’ views on GP services since the implementation of the new contract.^12^ The aim of this study was therefore to qualitatively explore the views of patients living in different settings (urban high deprivation, urban affluent/mixed, and remote and rural) on the changes in general practice in Scotland since the inception of the new GP contract.

**Table:** How this fits in

The Scottish Government formalised a new Scottish GP contract in April 2018, which aimed to improve quality of care through expansion of the primary care multidisciplinary team (MDT), thus enabling GPs to spend more time as expert medical generalists and supporting patients with complex needs. The knowledge and views of patients affected by these recent changes in general practice is largely unknown. This qualitative study with 30 primary care patients revealed ongoing difficulties regarding consultations with GPs across many areas—notably access, limited face-to-face GP appointments, short consultations, and lack of continuity of care (and its impact on the GP–patient relationship)—and although they were happy to consult MDT staff for specific problems, this was not felt to be a substitute for GP consultations about serious health concerns. These issues were especially concerning for patients with multiple complex problems from deprived areas and suggest that the expert generalist role of the GP is not being adequately supported by the new contract, especially in deprived areas.

## Method

This research is presented using the Standards for Reporting Qualitative Research framework^13^ and was conducted and reported in accordance with the Consolidated Criteria for Reporting Qualitative Research.^14^

### Study design

Qualitative methods were used to explore the views of patients consulting Scottish GPs by means of in-depth semi-structured interviews.

### Sampling and recruitment

This study is part of an ongoing programme of funded research, led by one of the authors on primary care transformation in Scotland. As part of this broader programme, 12 GP clusters were recruited across three Scottish health boards (out of a total of 14 regional health boards) for qualitative and quantitative evaluation.^15^^,^^16^ The participating regional health boards were selected to provide a variety of population characteristics, including urban areas of high deprivation (health board one), urban affluent/mixed (health board two), and remote and rural populations (health board three). Patients who were interested in potentially taking part in the qualitative study were identified from a postal survey of patients (also part of the programme of research).^16^ Sampling was designed to ensure that equal numbers of patients were recruited from the three health boards, and that there was a mix of characteristics according to age, sex, deprivation, and number of long-term conditions. Further details of the overall sampling frame, the patient survey, and the sampling of patients for interview are given in the Supplementary Information S1.

### Data collection

One-to-one telephone interviews with patients, lasting approximately 40–60 min, were conducted by two of the authors between November 2022 and January 2023, which were audio recorded and transcribed verbatim. The interview topic guide included their knowledge of the new GP contract, the impact of the pandemic on the services provided by their GP practices, their experiences of access, GP consultations, and consultations with new MDT staff. They were also asked what needed to be done to improve primary care services in the future (see Supplementary Information S1 for interview schedule). The topic guide was not piloted as such, but its use was discussed by three of the authors after the first few interviews on an iterative basis and was felt to be thorough and easily understood by participants in the three sites.

### Patient and public involvement group

A patient and public involvement (PPI) group consisting of four members participated in all steps in the research and were a core part of the team for this study and the entire research programme. More details are given in the Supplementary Information S1.

### Data analysis

Three of the authors developed initial codes based on independent analysis of three interview transcripts (one patient from each health board) and reached agreement on the initial coding frame through discussion. One author is an experienced qualitative researcher and another author is a mixed-methods researcher with 30 years of experience. A further author is a junior clinical researcher and was mentored by the aforementioned authors. Further details of their training and experience are given in Supplementary Information S1. Transcripts were coded using NVivo (version 12 Pro) by two of the authors. The phases of thematic analysis outlined by Braun and Clarke^17^^,^^18^ were conducted by the same two authors (supported by the other author) in the recommended six steps: familiarisation with the data; generation of initial codes; searching for themes; reviewing themes; defining and naming themes; and producing the final report. Emergent themes were discussed with the members of the PPI group established for this programme of research, as well as the other members of the wider research team. After coding and thematic analysis were complete, the two authors then specifically looked for variation between the accounts of patients from the three sites (patients living in urban deprived areas, urban affluent/mixed areas, or remote and rural areas). Emergent findings were discussed with the research team, including the PPI group. This study reported the key findings with examples of quotations below, but further quotations are given in the Supplementary Information S1.

## Results

Table 1 shows the characteristics of the patients who participated in the interviews. Across the three health board sites, this study achieved a similar range of ages and a 1:1 ratio of males to females. All deciles of deprivation were represented and reflected the expected differences across sites. Most of those interviewed had multimorbidity, but each site also included patients who did not.

**Table 1.** Patient characteristics

**Health board 1**	**Age, years**	**Sex**	**SIMD^a^**	**Chronic illnesses, *n***
P1 Urban deprived	39	Female	4	4
P2 Urban deprived	78	Female	2	5
P3 Urban deprived	63	Male	1	4
P4 Urban deprived	65	Female	2	8
P5 Urban deprived	74	Male	3	2
P6 Urban deprived	55	Male	1	2
P7 Urban deprived	66	Male	2	4
P8 Urban deprived	61	Female	5	1
P9 Urban deprived	41	Female	2	1
P10 Urban deprived	28	Male	4	2
**Health board 2**	**Age**	**Sex**	**SIMD**	**Chronic illnesses, *n***
P11 Urban affluent/mixed	78	Male	9	6
P12 Urban affluent/mixed	31	Female	3	4
P13 Urban affluent/mixed	65	Female	8	5
P14 Urban affluent/mixed	79	Male	10	4
P15 Urban affluent/mixed	75	Male	10	4
P16 Urban affluent/mixed	52	Female	7	4
P17 Urban affluent/mixed	64	Female	9	6
P18 Urban affluent/mixed	76	Female	10	1
P19 Urban affluent/mixed	30	Male	10	1
P20 Urban affluent/mixed	77	Male	10	1
**Health board 3**	**Age**	**Sex**	**SIMD**	**Chronic illnesses, *n***
P21 Remote and rural	81	Female	4	9
P22 Remote and rural	68	Female	4	4
P23 Remote and rural	73	Female	6	7
P24 Remote and rural	76	Male	5	3
P25 Remote and rura	57	Female	5	4
P26 Remote and rural	66	Male	3	6
P27 Remote and rural	60	Male	5	2
P28 Remote and rural	67	Male	4	1
P29 Remote and rural	23	Female	5	1
P30 Remote and rural	74	Male	4	0

a *1 = most deprived, 10 = most affluent. P = participant. SIMD = Scottish Index of Multiple Deprivation.*

Patients were generally unaware of the new 2018 Scottish GP contract and thought that recent changes in services were predominantly a response to the COVID-19 pandemic. Patients understood that GP practices had to adapt their services during the pandemic, but most felt that GP services had not returned to pre-pandemic standards:

*‘Do I remember hearing or seeing any information that they were introducing these sorts of changes to GP services? No, I thought that all came about after the pandemic. No, I don’t remember seeing anything about these changes… I thought it would just all go back to normal, when everything else went back to normal, after the pandemic but they didn’t… Since the pandemic, they’ve still got the same amount of GPs, and I don’t know what’s happening, are they all changing to phone calls now?’*

(P4, urban deprived — Scottish Index of Multiple Deprivation [SIMD] 2)

The key themes identified were: 1) access, 2) type of GP consultation, 3) time, 4) continuity of care and the GP–patient relationship, 5) views on being treated by new MDT staff, and 6) patients’ wishes for future improvement in primary care services. These themes are explored below.

### Theme one: access

#### Getting an appointment

Patients described a range of appointment booking and enquiry systems at their GP practice. Most felt that booking an appointment was more difficult than before the pandemic. The biggest initial problem was accessing the surgery receptionists to book an appointment due to the long telephone queuing waits in the morning and the fact that appointments were often filled by the time their call was answered:

*‘Now the system is that you have to phone at a given time. They only open at 8:45am and it is a recorded message. They will say you are in a queue, position 22. They have only just opened! I think there is only two phone lines at the front desk so I do not like that system. I don’t know whether it’s designed to put you off getting appointments. I am not a great fan.’*

(P7, urban deprived — SIMD 2)

*‘The telephone service, I think, could be revamped. I do not think it’s the best, to be fair. Getting through to practice receptionists is not always easy. The biggest problem is when you phone the surgery, there’s so many buttons. There is not always the option just to speak to a receptionist about what you want.’*

(P17, urban affluent/mixed — SIMD 9)

#### Signposting by reception staff

Once patients got through to the practice on the phone, it was reported to be standard practice now to be asked about their health issue by receptionist staff. While most patients accepted that this was the ‘new normal’, more than half felt uncomfortable with this in terms of privacy and confidentiality, especially if reception staff lived locally:

*‘Oh, I just hate it. I do not like it. Don’t get me wrong, I can phone a receptionist who’s been there a long time and say, “I need to see a GP because I can hardly breathe”. I’ll get an appointment. There are other times I do not want to say why I want to be there. I just need to see a doctor. The receptionist will say, “well, I really need to get an idea”. But I don’t want to tell you. Then they get quite stroppy… I know some of those girls on reception. Some of them know my family. I am not discussing such personal things with them.’*

(P4, urban deprived — SIMD 2)

*‘If it was something quite personal, or hard to speak about, it was hard to explain it to the receptionist. It felt quite uncomfortable. I was trying to be brief because I did not want to go into detail. They would say,* “what do you mean?” *And I didn’t want to explain it… I would rather speak to the doctor about it because it felt more private.’*

(P29, remote and rural — SIMD 5)

### Theme two: type of GP consultation

Almost all patients expressed a preference for having face-to-face GP consultations. They felt that such consultations gave a more personal connection with the GP than telephone consultations:

*‘I do prefer to go in face-to-face* [with a GP]*. It’s more of a connection. I can explain myself more… It’s like the GP helps me tell them what’s going on with me. On the phone, it’s not so personal.’*

(P6, urban deprived — SIMD 1)

Many patients believed that face-to-face consultations improved the quality of the clinical assessment. For some this was because a physical examination could take place:

*‘I do not like telephone consultations. I like to see the doctor face-to-face. What if you need* [to be] *examined?’*

(P4, urban deprived — SIMD 2)

Face-to-face consultations with the GP were regarded as especially important by older patients and those from deprived areas, who often had the most complex healthcare needs. Some patients were broadly satisfied with telephone consultations, especially if they had a pre-existing good relationship with their GP and/or had previously had a recent face-to-face consultation to make a diagnosis and/or to discuss managing their health issues. Overall, however, the perceived rationing of face-to-face GP appointments and the increase in GP phone consultations were seen as a backward step.

### Theme three: time

Over half (*n* = 17) of the patients interviewed felt they had enough time in their GP consultations but were aware of greater time pressures on GPs since the pandemic. Satisfaction with consultation length was more common in the urban affluent and the remote and rural patients than in urban deprived areas, where more patients were dissatisfied with GP consultation length:

*‘Most of the time it is sufficient time with the GP. You never feel as if you are being hassled out the door.’*

(P21, remote and rural — SIMD 4)

*‘My time with the GP, I never feel rushed. But in almost every occasion, I feel that the poor old GP is under a lot of* [time] *pressure. I do get the distinct impression the GPs are under a lot of pressure. They seem up against it time-wise.’*

(P15, urban affluent — SIMD 10)

*‘It’s usually about 10–15 minutes with the GP… You can’t really elaborate properly on what the problem is. In an ideal world, I’d like 20–25 minutes… I just always have that feeling at the back of my mind that they are really trying to get you out the door as fast as possible*.’

(P10, urban deprived — SIMD 4)

Of the patients who were dissatisfied with the consultation length with GPs, some were especially critical of the lack of time in telephone consultations:

*‘With the telephone consultations, that is where I feel more rushed. When you’re on the phone, after them I tend to say, “oh, I wish I’d said this, I wish I’d said that” … They’ve not much time with a telephone appointment, so you’re a bit more rushed. That is why I like to see the doctor face-to-face.’*

(P4, urban deprived — SIMD 2)

### Theme four: continuity of care and the patient–GP relationship

Three-quarters of patients interviewed reported that, up until the pandemic, they had a preferred GP that they would preferentially consult with because of the established relationship and trust in confiding in them. Patients associated continuity of care and a positive GP*–*patient relationship with greater satisfaction and better health outcomes. This was apparent among patients from all areas, but was especially emphasised by people from the most deprived areas, and those with the most complex health needs:

*‘I like to see the same GP. It’s about trust… I can tell my GP anything. I told her about the trauma I experienced as a child. I never told nobody before about that. She made me feel so comfortable that I was able to tell her and express how I was feeling… we worked through it together and I got the help I needed… It’s about building relationships… Every time you see a new GP it is like starting from the beginning again.’ (*

P6 urban deprived — SIMD 1)

Many patients commented on the negative impact that post-pandemic GP services were having on continuity of care and the GP*–*patient relationship. Patients with very complex care needs, especially those with mental health problems, were reticent about sharing their concerns with a GP they didn’t know well and (as long as their health issue didn’t require immediate attention) would prefer to wait several weeks to see their preferred GP:

*‘I have a preferred GP, one of the female doctors, I really like her. She says to me when I go in, “how are you”, and I say, “I am fine”. Then she says, “no, how are you really?”... With the newer GPs, I don’t feel they know me well enough to talk about everything. So, I just wait until I see that one GP… Yes, that’s exactly it, that GP understands me better.’*

(P4, urban deprived — SIMD 2)

### Theme five: consultations with new MDT staff

Most patients had not heard in advance about the expansion of the MDT team, but most said that they were happy to see MDT staff, especially if the GP then thought they would benefit from such a consultation:

*‘If I went to the GP tomorrow with a wrist injury and they wanted to refer me to a practice physio, or the receptionist said, we’ll book you in with the practice physio, I’d be happy. To me, that would make total sense… It also takes pressure off the GPs.’*

(P25, remote and rural — SIMD 5)

Almost all the patients interviewed had experienced an MDT consultation of some type and most were positive, partly because consultations with MDT staff seemed easier to book than with GPs and were usually much longer:

*‘I had started some new medication for my anxiety and I was having side effects. I called the GP reception. They said I could either talk to the pharmacist ASAP or wait to speak with the GP. My initial reaction was, “I want to talk to my GP as they had prescribed it”. But I went with the pharmacist. Ultimately, getting that answer from the pharmacist was helpful and what I needed. I was happy. I am learning about the NHS as it is now. I’m rolling with it. That is what I should be doing*—*this was a pharmacy issue.’*

(P19, urban affluent — SIMD 10)

*‘Well, the positive one for me was getting that physio appointment. The GP arranged that. I didn’t need to then wait for another GP appointment and I didn’t have to go elsewhere. It was all in the one place. The physio told me that it was not my knee that was giving me all that bother. He said it is your hip. Now I am on the waiting list to get a replacement hip.’*

(P2, urban deprived — SIMD 2)

However, all patients still expected and wanted to see a GP for health concerns they (the patients) considered to be potentially serious:

*‘I think sometimes you need to see a GP right away… I think you should be listened to carefully about your symptoms before you’re referred to somebody other than the GP. I still would rather value a GP’s opinion than any of the other professionals. It does depend very much on what your query is but if you’re feeling really ill, I think you need to have a GP examining you… I still feel that the GPs are more lengthily trained. The GPs’ training, the GP’s diagnosis, I’d have more confidence in that than any other professional.’*

(P18, urban affluent/mixed — SIMD 10)

### Theme six: improvements required in general practice

When asked what improvements they would like to see now at their GP practice, over two-thirds of patients said quicker access to GPs, more face-to-face GP consultations, and longer consultations with GPs. This was across all patient backgrounds but was especially emphasised by the most deprived patients with complex problems:

*‘Definitely, quicker appointments and face-to-face. I just prefer to physically see my GP. And, when I get one, I just don’t think 10 or 15 minutes is long enough. You used to go to the doctor, you sat, you were at ease. I feel that I’m rushing when I go to the GP now. Also, I don’t like waiting so long for the appointment. When I’m ill I want to know what’s wrong with me or my mind starts running riot.’*

(P2, urban deprived — SIMD 2)

*‘When the GPs can* [physically] *see you, they know from seeing you for years roughly how you are, also your mental health. They can pick up things on* [physically] *seeing you. When care is dotted around different people seeing you, some of that knowledge can get lost. That worries me. You can have something on paper* [patient records] *but that is not the same as knowing a wee bit about the family history. You’ve got a bit more knowledge with that.’*

(P21, remote and rural — SIMD 4)

Given the significant increase in telephone consultations, which most patients acknowledged were likely to continue, a better system of organising remote consultations was called for:

*‘With telephone consultations, what can be difficult is it depends where you are. I answered it once when on a busy bus and you just think, “oh, no, you had to phone now”. There are a hundred things I wanted to ask the GP, but I can’t because I’ve waited 3 weeks for this appointment and you phone now when I’m on a busy bus. So, yes, I think it does become difficult because it depends where you are when you answer that call.’*

(P16, urban affluent — SIMD 7)

Finally, many patients were very aware of the workforce issues in general practice:

*‘Increase the GP workforce. Get another couple of GPs into the practices so that the ones who are there don’t feel so stressed out of their heads. I just think we need to get more GPs who are motivated, are going to stay on in Scotland, and feel they’re doing a good job and they’re appreciated.’*

(P15, urban affluent — SIMD 10)

## Discussion

### Summary

In this qualitative study of patients who had recently consulted a GP, the authors’ found that most were unaware of the 2018 GP contract in Scotland and attributed recent changes in general practice to the COVID-19 pandemic. Their concerns included difficulties in access (getting through on the phone and then being signposted by reception staff), the lack of availability of face-to-face GP consultations, the short duration of GP consultations, and the lack of continuity of GP care. The negative impact of these factors on the GP–patient relationship—which they highly valued—was also a prominent concern. Most patients spoke positively about consultations with MDT staff but still wanted to see a known GP for serious health concerns. These issues were especially concerning for patients with multiple complex problems from deprived areas.

### Strengths and limitations

Strengths of the study were the involvement of the four members of the PPI group in all stages of the study and the inclusion of patients across three diverse health board areas, enabling the consideration of views from patients living in urban deprived areas, urban affluent/mixed areas, and remote and rural areas of Scotland, using the same sampling frame from the authors’ linked work.^8^^,^^15^^,^^16^ However, as this study recruited from patients who had taken part in the authors’ patient survey (see Supplementary Information S1), it could be that the patients were more motivated to be interviewed because of strong views, positive or negative, than those who did not take part. However, apart from higher levels of multimorbidity (in line with the study’s purposive sampling strategy), there were no differences in any of the consultation quality measures between those interviewed and the rest of the survey sample (see Supplementary Information S1), which gives some reassurance that the patients interviewed were not ‘outliers’ in terms of their views on consultation quality.

The interviews were conducted between November 2022 and January 2023, months that are traditionally busy in general practice, which could have conceivably coloured patients views on access and consultation length if they consulted during this period. This is unknown as the authors did not collect information on this (the authors identified patients who had consulted a GP in the previous 30 days in the week beginning 22 August 2022, who were then sent the survey. See Supplementary Information S1 for full details).

This study’s findings were from interviews with only 30 patients (10 from each setting). However, the authors are confident that data saturation was reached, as no new themes emerged in the latter interviews. Additionally, the aim of qualitative research is to explore issues of context and depth, rather than generalisability and the authors were able to recruit patients for interviews in line with the pre-specified purposive sample.

### Comparison with existing literature

As far as the authors are aware, this is the first study to seek patients’ qualitative views on the new Scottish GP contract. The finding that patients were unaware of the new contract was in line with international evidence that patients are rarely consulted about proposed reforms or included in the evaluation of such reforms,^9^ despite the evidence that patient engagement can meaningfully inform policies and enhance service delivery and governance.^19^ Expansion of the MDT is the most common policy response by high income countries to primary care transformation.^9^ The current finding that patients with multimorbidity were the least comfortable with these changes agreed with studies in other high-income countries.^20^^,^^21^ A recent report from England also found similar findings to this study regarding patients living in deprived areas.^22^

Many of the concerns raised by patients are supported by the authors’ aligned research, including the views of national stakeholders and GP cluster leads,^15^ other GPs and MDT staff,^8^ and the authors’ recent patient survey that found lower satisfaction and outcomes in patients with complex problems living in deprived urban areas compared with those living in urban affluent or remote and rural settings^16^ (similar to previous findings almost 2 decades ago).^23^ This study’s findings also agreed with a recent Scottish Government survey.^12^ The preference for face-to-face consultations, particularly among those from the most deprived areas with complex and mental/physical health needs, echoes other recent research in the UK,^24^^–^^26^ which is concerning given that patients in deprived areas receive more telephone consultations than face-to-face compared with patients in other settings.^16^^,^^27^

### Implications for research and practice

Although most patients in this study acknowledged the unprecedented demand facing GP services post-pandemic, their anxieties about reduced face-to-face access to GPs and the disruption to relational continuity of care have important implications. Personalised, relationship-based holistic care is the hallmark of general practice^28^ and empathic, person-centred GP consultations improve patient enablement,^29^ health outcomes,^30^ and mortality.^31^ Similarly, continuity of care in general practice has been shown to reduce unscheduled care, unplanned hospital admission and mortality.^32^ Providing such holistic care in general practice is challenging anyway, but especially so when consulting remotely.^33^ The added burden of the inverse care law makes such care even more challenging in areas of deprivation,^16^^,^^23^ where patients develop multimorbidity some 10–15 years earlier than those living in affluent areas.^1^

A key aim of the 2018 GP contract was to reduce workload so that GPs could enhance their expert generalist role and focus more time on patients with complex healthcare needs. Such needs are most prominent in patients with complex multimorbidity, which includes those in later life and those at a younger age living in deprived areas.^34^ From the current study, and the authors’ linked research, there is no evidence that this is happening at scale.^8^^,^^15^^,^^16^ Clearly the pandemic has had a major negative impact, but progress in the implementation of the contract was reported to be slow prior to the pandemic.^8^^,^^15^^,^^35^^,^^36^ It is concerning that most of the challenges (other than the pandemic) were predicted by senior primary care stakeholders in 2016.^37^

It is clear some of the issues raised by patients were not due to the new GP contract per se, but rather to changes imposed during the pandemic that remain in place. Whereas signposting by reception staff and expansion of the MDT are clearly part of the new GP contract, remote consulting is not, and was simply a response to the pandemic,^38^ which has (to a variable degree) persisted as GPs struggle to cope with increased workload post-pandemic.^39^ Further quantitative research is required to substantiate the authors’ qualitative studies on primary care transformation in Scotland, and such work is currently underway.

The patients interviewed in this study were mainly unaware of the new GP contract. Although most were accepting of the increased role of MDT staff in general practice, face-to-face consultations with a known GP, together with more time and continuity of care were high priorities, especially for health concerns that they considered potentially serious. These issues were especially concerning for patients with multiple complex problems, particularly those from deprived areas. More support to reduce GP workload to allow such care is urgently needed if the vision of the 2018 Scottish GP contract is to be realised.
